# Supplementary material for: A Cellulose-Derived Nanofibrous MnO2-TiO2-Carbon Composite as Anodic Material for Lithium-Ion Batteries
Source: Materials (Basel). 2021 Jun 20;14(12):3411. doi: 10.3390/ma14123411 (PMC8234856; doi:10.3390/ma14123411)
Supplement: Supplementary file 1 [file materials-14-03411-s001.zip › materials-1261241-supplementary.pdf]

materials

Supplementary Materials: A Cellulose-Derived Nanofibrous MnO_2-TiO_2-Carbon Composite as Anodic Material for Lithium-Ion Batteries

Shun Li [1,2,*], Ming Yang [1], Guijin He [1], Dongmei Qi [3] and Jianguo Huang [1,*]

[1] Department of Chemistry, Zhejiang University, Hangzhou 310027, China; 21937064@zju.edu.cn (M.Y.); guikinghe@zju.edu.cn (G.H.)
[2] School of Engineering, Zhejiang Agriculture and Forestry University, Hangzhou 311300, China
[3] Analysis Center of Agrobiology and Environmental Sciences, Zhejiang University, Hangzhou 310027, China; qidongmei@zju.edu.cn
* **Correspondence:** smoothlee2013@zju.edu.cn (S.L.); jghuang@zju.edu.cn (J.H.); Tel.: +86-571-8795-1202 (J.H.)

Citation: Li, S.; Yang, M.; He, G.; Qi, D.; Huang, J. A Cellulose-Derived Nanofibrous MnO_2-TiO_2-Carbon Composite as Anodic Material for Lithium-Ion Batteries. *Int. J. Environ. Res. Public Health* **2021**, *14*, 3411. https://doi.org/10.3390/ma14123411

Academic Editor: Halina Kaczmarek

Received: 31 May 2021
Accepted: 17 June 2021
Published: 20 June 2021

Publisher's Note: MDPI stays neutral with regard to jurisdictional claims in published maps and institutional affiliations.

Copyright: © 2021 by the authors. Licensee MDPI, Basel, Switzerland. This article is an open access article distributed under the terms and conditions of the Creative Commons Attribution (CC BY) license (http://creativecommons.org/licenses/by/4.0/).

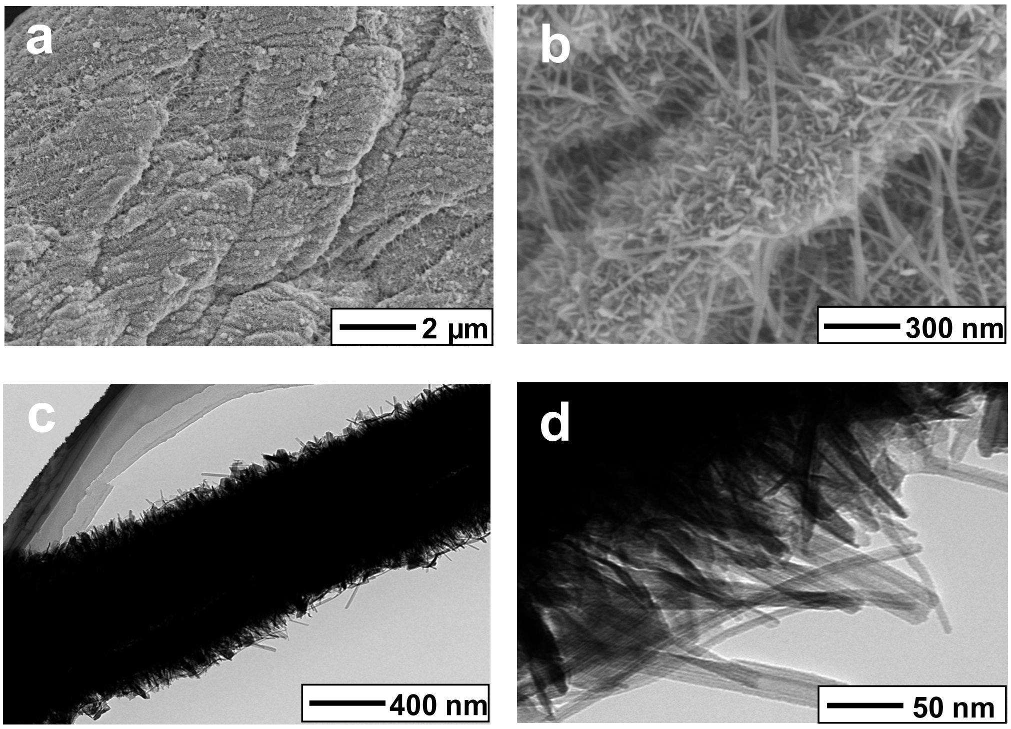

Figure S1. Electron micrographs of the nanofibrous MnO_2-TiO_2-carbon-37.81% composite derived from the natural cellulose substance. (**a**) SEM image of the MnO_2-TiO_2-carbon-37.81% composite, (**b**) SEM image of an individual composite nanofiber isolated from the assemblies, (**c**) and (**d**) the TEM images of an individual MnO_2-TiO_2-carbon-37.81% nanofiber at different magnifications.

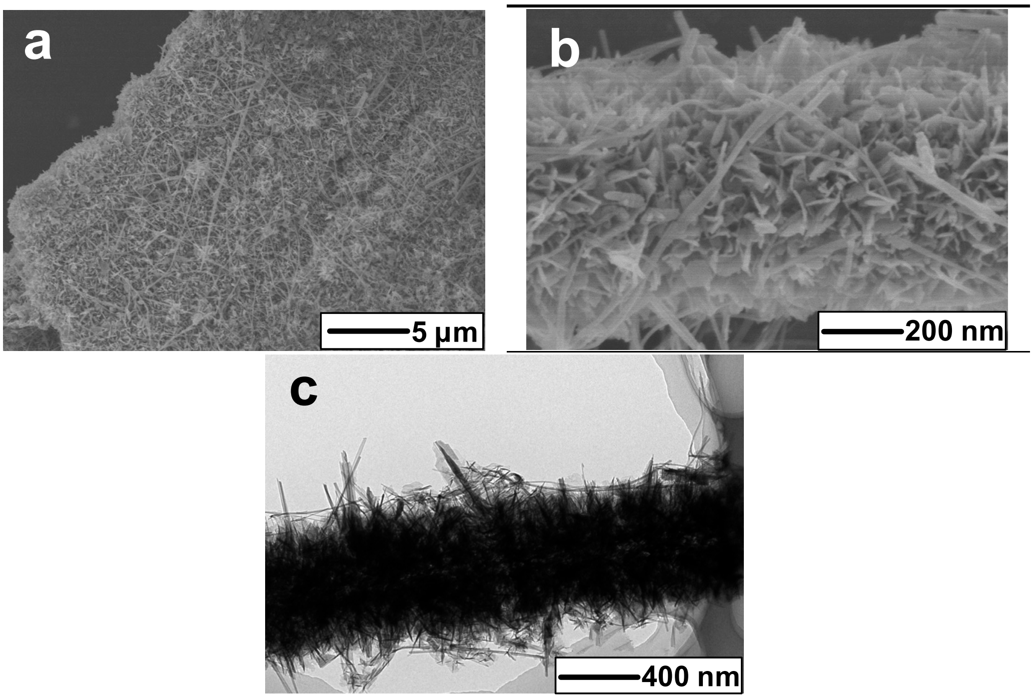

Figure S2. Electron micrographs of the nanofibrous MnO_2-carbon-33.30% composite derived from the natural cellulose substance. (**a**) SEM image of the MnO_2-carbon-33.30% composite, (**b**) SEM and (**c**) TEM images of an individual composite nanofiber.

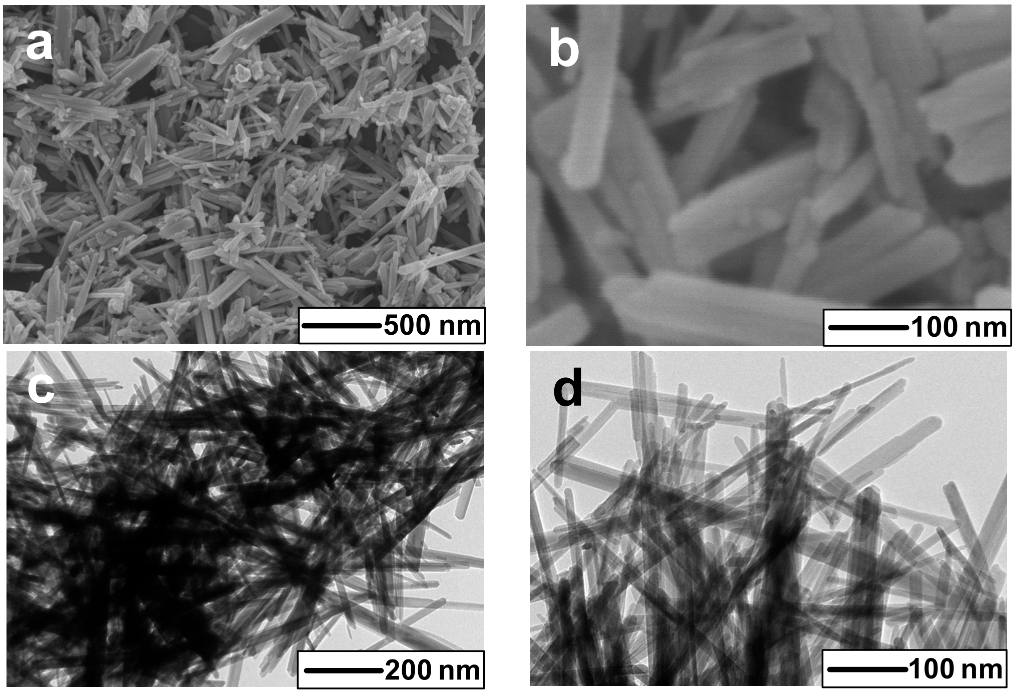

Figure S3. (**a**), (**b**) SEM and (**c**), (**d**) TEM micrographs of the MnO_2-NPs materials.

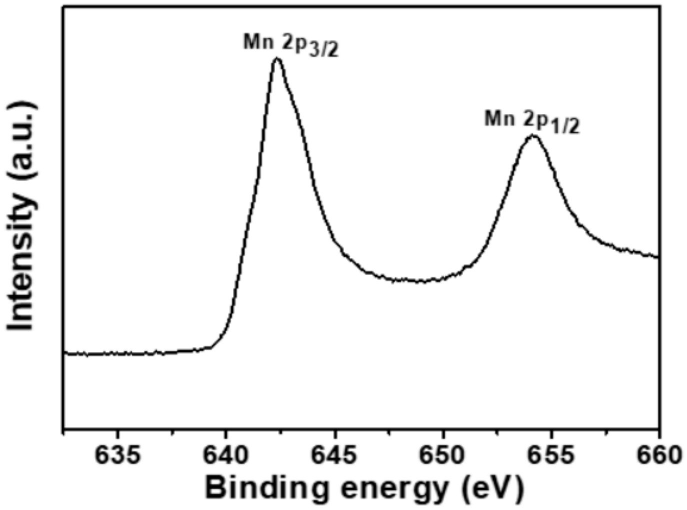

Figure S4. The high-resolution XPS spectra of Mn 2p regions of the MnO_2-TiO_2-carbon-37.81% composite.

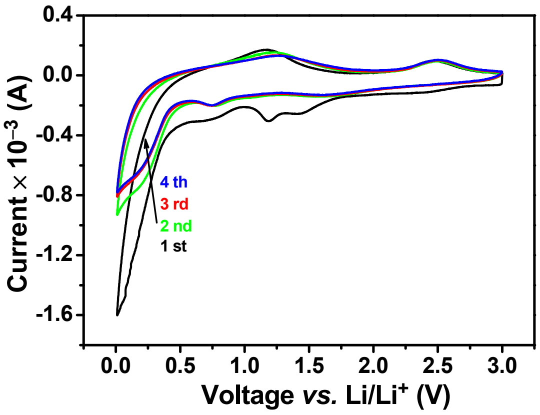

(**a**)

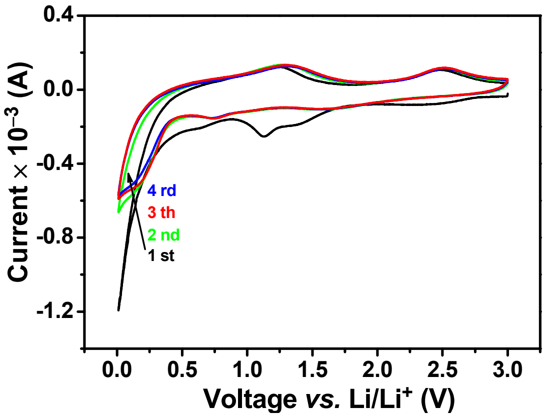

(**b**)

Figure S5. Cyclic voltammetry curves of (**a**) MnO_2-TiO_2-carbon-37.81% and (**b**) MnO_2-carbon-33.30% electrodes tested in the initial four charge/discharge cycles at a scan rate of 0.2 mV s^{-1} between 0.01 and 3 V (*versus* Li/Li^+).

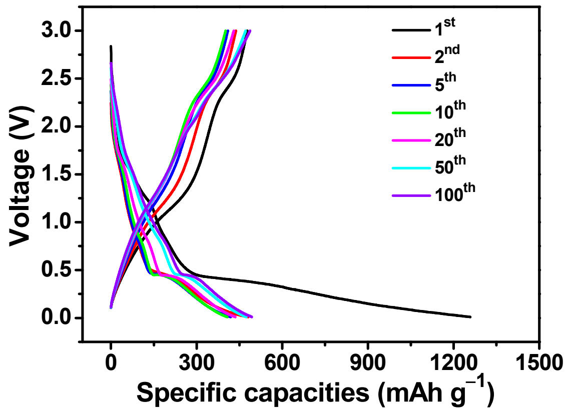

(**a**)

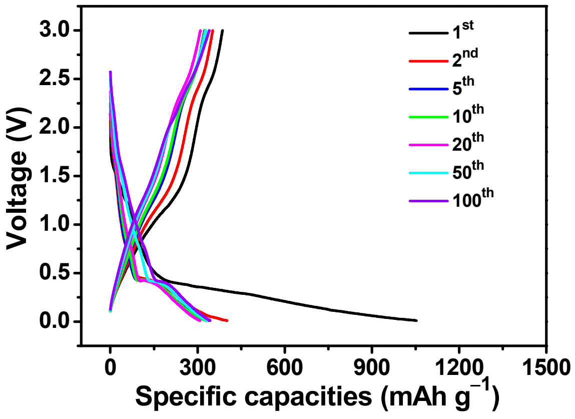

(**b**)

Figure S6. Galvanostatic charge/discharge profiles of (**a**) MnO_2-TiO_2-carbon-37.81% and (**b**) MnO_2-carbon-33.30% anode materials measured at a current density of 100 mA g^{-1} between 0.01 and 3.0 V.

Table S1. Equivalent circuit parameters obtained from fitting the experimental impedance spectra of the MnO_2-TiO_2-carbon-47.28%, MnO_2-carbon-33.30 and TiO_2-carbon nanocomposite electrodes.

Samples	R_s (Ω)	R_{ct} (Ω)
MnO_2-TiO_2-carbon-47.28%	8.719	46.32
MnO_2-carbon-33.30	12.34	155
TiO_2-carbon	12.71	231.7

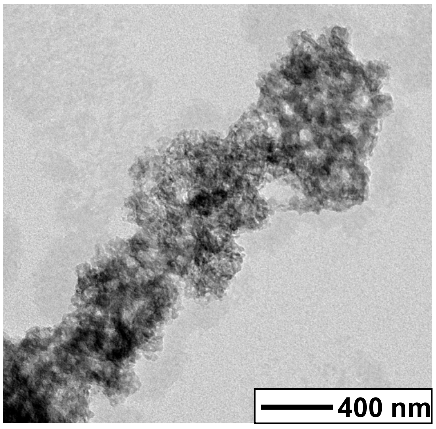

Figure S7. TEM image of the MnO_2-TiO_2-carbon-47.28% anode material after 200 charge/discharge cycles.
